# Identification of *ireA*, *0007, 0008,* and *2235* as TonB-dependent receptors in the avian pathogenic *Escherichia coli* strain DE205B

**DOI:** 10.1186/s13567-020-0734-z

**Published:** 2020-01-23

**Authors:** Zhonghua Zhang, Shan Jiang, Yun Liu, Yu Sun, Peixin Yu, Qianwen Gong, Hang Zeng, Yihao Li, Feng Xue, Xiangkai Zhuge, Jianluan Ren, Jianjun Dai, Fang Tang

**Affiliations:** MOE Joint International Research Laboratory of Animal Health and Food Safety, Key Laboratory of Animal Bacteriology, Ministry of Agriculture, College of Veterinary Medicine, Nanjing Agricultural University, Nanjing, 210095 China

## Abstract

Avian pathogenic *Escherichia coli* (APEC), a pathotype of extraintestinal pathogenic *E. coli*, causes one of the most serious infectious diseases of poultry and shares some common virulence genes with neonatal meningitis-associated *E. coli*. TonB-dependent receptors (TBDRs) are ubiquitous outer membrane β-barrel proteins; they play an important role in the recognition of siderophores during iron uptake. Here, in the APEC strain DE205B, we investigated the role of four putative TBDRs—*ireA*, *0007*, *0008*, and *2235*—in iron uptake. Glutathione-S-transferase pulldown assays indicated that the proteins encoded by these genes directly interact with TonB. Moreover, the expression levels of all four genes were significantly upregulated under iron-depleted conditions compared with iron-rich conditions. The expression levels of several iron uptake-related genes were significantly increased in the *ireA*, *0007*, *0008*, and *2235* deletion strains, with the upregulation being the most prominent in the *ireA* deletion mutant. Furthermore, iron uptake by the *ireA* deletion strain was significantly increased compared to that by the wild-type strain. Moreover, a *tonB* mutant strain was constructed to study the effect of *tonB* deletion on the TBDRs. We found that regardless of the presence of *tonB*, the expression levels of the genes encoding the four TBDRs were regulated by *fur*. In conclusion, our findings indicated that *ireA*, *0007*, *0008*, and *2235* indeed encode TBDRs, with *ireA* having the most important role in iron uptake. These results should help future studies explore the mechanisms underlying the TonB-dependent iron uptake pathway.

## Introduction

Avian pathogenic *Escherichia coli* (APEC), a pathotype of extraintestinal pathogenic *E. coli* (ExPEC), causes serious infectious diseases in poultry [1, 2]. Different serotypes of APEC cause local or systemic infectious diseases in poultry, including respiratory infections, sepsis, polyserositis, coligranuloma, cellulitis, yolk sac infection, omphalitis, and swollen head syndrome, resulting in significant economic losses to the poultry industry [3]. Strains of APEC and neonatal meningitis-associated *E. coli* (NMEC, a subpathotype of ExPEC), the latter of which causes infections in humans, reportedly share some common virulence genes [4–6]. It is thus particularly important to study the genes encoding virulence factors in APEC strains. These strains contain several virulence-associated genes that encode various virulence factors, including adhesins (*fimC*, *ompA*, *papC*), invasins (*ibeA*), avian haemolysins (*hlyF*), serum survival proteins (*iss*, *ompT*), and siderophores (*iutA*, *fyuA*, *iroN*); moreover, they show the presence of a pathogenicity island [7–9].

Iron is a vital micronutrient that regulates enzyme activity and metabolism. This element plays a key role in basic cellular processes, such as cellular respiration, DNA replication, and electron transport and is accordingly essential for bacterial survival in host tissues [10–12]. Furthermore, iron is a necessary growth factor for bacteria and is reportedly involved in the expression of bacterial virulence factors [13]. Iron uptake factors evidently play a pivotal role in *E. coli* growth and pathogenesis. Bacteria employ different strategies to absorb iron from their environment, including siderophore-mediated iron uptake, which occurs on the cell surface [14]. In addition, bacteria either excessively reduce the external pH or dissolve iron oxide to meet their iron requirements by reducing ferric iron to a relatively soluble ferrous form. Another common strategy is to synthesize and secrete iron chelators, such as siderophores, as intracellular iron (Fe^2+^) is rarely found in natural conditions, and Fe^2+^ can be readily oxidized to Fe^3+^ in the presence of oxygen and water [15]. Siderophores then combine with the available iron (Fe^3+^) to form an iron–siderophore complex, which binds to specific receptor proteins on the bacterial cell surface, consequently entering cells via the TonB-dependent transport system, followed by iron release. Siderophore-mediated ferric uptake requires specific outer membrane (OM) receptors, such as FhuA, FecA, and FepA, which reportedly exist in *E. coli* [16]. These OM receptors share the same structural properties and acquire energy by coupling with TonB proteins located in the inner membrane, and they are referred to as TonB-dependent receptors (TBDRs) [17].

TBDRs are known to actively transport ferric–siderophore complexes in Gram-negative bacteria, and they also transport diverse antibiotics, vitamins, nickel complexes, and carbohydrates [18–20]. Transporters involved in iron uptake have very strict siderophore selectivity. A strong correlation exists between the amount of iron and siderophores that bacteria can use and the number of genes encoding iron-regulated TBDRs [19]. In simple terms, iron depletion triggers the upregulation of genes encoding TBDRs.

In this study, upon analysing the whole genome of the APEC strain DE205B, we identified six putative TBDRs; however, the construction of two mutants failed. Thus, we eventually investigated the roles of four putative TBDRs—*ireA*, *0007*, *0008*, and *2235*—in iron uptake.

## Materials and methods

### Bacterial strains, plasmids, and growth conditions

The bacterial strains and plasmids used in this study are listed in Table 1. The APEC strain DE205B (O2:K1), which was isolated from a duck with neurological symptoms, was previously determined to have no antibiotic (ampicillin, kanamycin, and chloramphenicol) resistance [21–25]. All the APEC strains used in this study were cultured in Luria–Bertani (LB, Oxoid, Thermo-Fisher, USA) or solid LB medium supplemented with 2% agar, followed by incubation in a shaker at 37 °C and 180 rpm unless otherwise stated. For culture of the mutant strain, appropriate antibiotics (ampicillin, 100 μg/mL; kanamycin, 50 μg/mL; chloramphenicol, 15 μg/mL) were added to the LB media. M9 medium was prepared as follows: 200 mL of 5 × M9 salt solution (12.8 g Na_2_PO_4_·7H_2_O, 3.0 g KH_2_PO_4_, 0.5 g NaCl, and 1.0 g NH_4_Cl) was mixed with 2 mL of 1 M MgSO_4_, 20 mL of 20% glucose, and 0.1 mL of 1 M CaCl_2_. This solution was then dissolved in 1000 mL double-distilled water and filtered through a 0.22-μm membrane. Fe (0.1 mM FeCl_3_·6H_2_O) was added or not added to M9 medium to establish iron-rich or iron-depleted conditions, respectively.

**Table 1:** **Bacterial strains and plasmids used in this study**.

Strain or plasmid	Characteristics	References
Strain		
DE205B	O2:K1, NalR	[21–25]
DE205BΔ*ireA*	*ireA* Deletion mutant strain	[21]
DE205BΔ*ireA*/ireA*	*ireA* Complemented strain	[21]
DE205BΔ*0007*	*0007* Deletion mutant strain	This study
DE205BΔ*0007*/0007*	*0007* Complemented strain	This study
DE205BΔ*0008*	*0008* Deletion mutant strain	This study
DE205BΔ*0008*/0008*	*0008* Complemented strain	This study
DE205BΔ*2235*	*2235* Deletion mutant strain	This study
DE205BΔ*2235*/2235*	*2235* Complemented strain	This study
DE205BΔ*tonB*	*tonB* deletion mutant strain	This study
DE205BΔ*tonB*/tonB*	*tonB* Complemented strain	This study
DH5a	Competent invitrogen cells	Vazyme
BL21	Competent invitrogen cells	Vazyme
Plasmid		
pKD46	Amp, expresses λ red recombinase	[26]
pKD4	Kan, template plasmid	[26]
pSTV28	Cm, expression using lac promotor	TaKaRa
pCP20	Cm, Amp, yeast Flp recombinase gene, FLp	[26]
pET32a	Amp, expresses a fusion fragment of the His tag	This study
pGEX-4t-1	Amp, expresses a fusion fragment of the GST tag	This study

### Construction of the mutant and complemented strains

For analysis of the role of the four TBDRs in iron uptake, three genes encoding TBDRs (*0007*, *0008*, *2235*) were knocked out, and the *ireA* mutant strain, which was previously constructed [21], was used in this study. A single mutant strain for each of the three genes (*0007*, *0008*, *2235*) was constructed using the Red homologous recombination method in *E. coli* [26]. Briefly, for generation of the *0007* mutant strain, a gene-targeting fragment containing a homologous arm on both sides of *0007* was amplified by PCR using the plasmid pKD4 as the template, which contains the phage λ Red system under the control of an arabinose promoter. Competent DE205B cells containing pKD46 were then prepared; l-arabinose was added to induce the expression of the phage λ Red system, and the target fragments were transformed by electroporation into DE205B to replace *0007* with the resistance gene. The recombinant strain was screened by growth on LB plates supplemented with kanamycin and identified by cross-PCR. Details of the primers (0007-F/0007-R, K1, K2) used for amplification are listed in Additional file 1. Next, the temperature-sensitive plasmid pCP20 was transformed into the recombinant strain to remove the resistance gene. Finally, pCP20 was removed by growth at 42 °C for 24 h to obtain the mutant strain DE205BΔ*0007* that did not show any resistance. For analysis of the effect of *tonB* deletion on the TBDRs, a *tonB* mutant strain was also constructed using the same method. Similarly, the *0008*, *2235*, and *tonB* mutant strains, namely, DE205BΔ*0008*, DE205BΔ*2235*, and DE205BΔ*tonB*, respectively, were obtained.

The construction of the complemented strain involved recovering the deleted gene. The genomic DNA of DE205B was used as the template to amplify *0007*, including its putative promoter gene. The PCR product was then purified and inserted into pSTV28, and the composite vector PSTV28-*0007* was transformed by electroporation into DE205BΔ*0007* to produce the complemented strain DE205BΔ*0007*/0007*. In the same manner, the complemented strains DE205BΔ*0008*/0008*, DE205BΔ*2235*/2235*, and DE205BΔ*tonB*/tonB* were obtained. The *ireA* deletion strain DE205BΔ*ireA* and the complemented strain DE205BΔ*ireA*/ireA* were constructed in an earlier study and used in this study as well [21].

### Growth curves

Growth curves of the wild-type (WT), mutant (DE205BΔ*ireA*, DE205BΔ*0007*, DE205BΔ*0008*, DE205BΔ*2235*) and complemented strains (DE205BΔ*ireA*/ireA*, DE205BΔ*0007*/0007*, DE205BΔ*0008*/0008*, DE205BΔ*2235*/2235*) cultured in LB, iron-depleted M9 and iron-rich M9 media were constructed. All strains were grown overnight in 5 mL of LB medium supplemented with antibiotics, as appropriate. The cultured strains were centrifuged at 5000 rpm for 10 min to remove the supernatant, and the cell pellets were washed twice with PBS containing 200 mM 2,2′-dipyridyl; the optical density at 600 nm (OD_600_) was adjusted to 1. Then, 100 μL of the suspension was inoculated into 50 mL of LB medium, and 1000 μL of this solution was inoculated into 50 mL of iron-depleted or iron-rich M9 medium. The OD_600_ of the LB culture was measured using a spectrophotometer (Philes, Nanjing, China) every hour for a total of 10 h and that of the iron-depleted and iron-rich M9 cultures was measured at 0, 4, 8, 12, 24, and 48 h [27].

### Gene expression analyses

As *ireA*, *0007* (GI: MN239889), *0008* (GI: MN239890), and *2235* (GI: MN239892) encode putative TBDRs, their expression levels were determined under iron-rich and iron-depleted conditions. DE205B was cultured to the mid-log phase in both types of M9 media, and the gene expression levels were determined using quantitative real-time PCR (qRT-PCR). Briefly, total RNA was extracted from bacteria grown under different culture conditions using a bacterial RNA kit (Omega Bio-Tek, Beijing, China) and reverse transcribed into cDNA using the PrimeScript™ RT reagent Kit (TaKaRa, Perfect Real Time, Japan) according to the manufacturer’s instructions. qRT-PCR was performed in a 20 μL reaction volume containing SYBR Green PCR Master Mix (Vazyme Biotech, Nanjing, China) and 0.1 μM primers (Additional file 1) specific to the iron uptake-related genes (*0007*, *0008*, *2235*, *ireA*, *fecA*, *fhuA*, *iutA*, *iroN1*, *iroN2*); the quantification data were analysed with ABI StepOne Software, version 2.3 (USA). For analysis of the effect of gene deletion on the ferric uptake system, the expression levels of iron uptake-related genes (*fecA*, *fhuA*, *fepA*, *fepC*, *feoB*, *fyuA*, *iutA*, *chuA*, *iroN1*, *iroN2*) were tested in LB medium. Total RNA was extracted from the WT, mutant (DE205BΔ*ireA*, DE205BΔ*0007*, DE205BΔ*0008*, DE205BΔ*2235*), and complemented (DE205BΔ*ireA*/ireA*, DE205BΔ*0007*/0007*, DE205BΔ*0008*/0008*, DE205BΔ*2235*/2235*) strains using the same method. For analysis of the effect of *tonB* on the expression levels of the genes encoding the four TBDRs, the gene (*0007*, *0008*, *2235*, *ireA*, *fecA*, *fhuA*, *fepA*, *iutA*, *feoB*) expression levels of the WT strain and DE205BΔ*tonB* cultured in iron-depleted M9 medium were examined. Primer details are listed in Additional file 1. *dnaE* was used as an internal reference gene [21, 22]. The relative gene expression levels were calculated using the 2^−△△Ct^ method; the values are expressed as percentages [28–30].

### Expression of the TBDRs

The pertinent gene fragments (*ireA*, *0007*, *0008*, *2235*) were inserted into the plasmid pET32a, and *tonB* was cloned into pGEX-4t-1. Proteins were expressed in BL21 (Vazyme Biotech) via addition of 1 mM isopropyl β-d-1-thiogalactopyranoside in the mid-log phase. The recombinant proteins IreA, 0007, 0008, and 2235 expressed in vitro carried a His tag, and TonB carried a glutathione-S-transferase (GST) tag. The primer details are listed in Additional file 1. The His-tag fusion proteins were purified by Ni-column chromatography, and the biological activity of the proteins in inclusion bodies was restored via a conventional method that involves using a urea-containing protein refolding solution (including 20 mM Tris–HCl, 1 mM GSH, 0.2 mM GSSG, 0.5 M NaCl). Finally, we concentrated and collected the putative TBDRs (IreA, 0007, 0008, 2235) with His tags by centrifugation in an ultrafiltration tube (10 kDa) at 4 °C. The final fusion proteins were thus obtained (34 kDa for 0007-His, 66 kDa for 0008-His, 95 kDa for 2235-His, and 93 kDa for IreA-His).

### GST pulldown assay

The functional domain of TonB (residues 150–239) is critical, as it directly interacts with the OM receptor [31]. As such, to further verify whether the four putative TBDRs directly interact with TonB, we performed GST pulldown assays. Cells expressing the GST fusion proteins were lysed using an ultrasonic cell disrupter system (Thermo Fisher Scientific, China). Unlysed cells and impurities were removed by centrifugation (5000 rpm, 10 min), and the supernatant was incubated with GST-binding beads (Enriching Biotechnology, Ltd., Shanghai, China) for 3 h, followed by stringent washing with PBS (2 mM KH_2_PO_4_, 2.6 mM KCl, 8 mM Na_2_HPO_4_, 136 mM NaCl) to minimize nonspecific binding. Next, the samples were incubated with the putative TBDRs (IreA, 0007, 0008, 2235) with His tags. Samples were collected after multiple washes with PBS to minimize nonspecific binding. Finally, protein separation was performed by SDS–PAGE on 12% protein gels (Warbio, Shanghai, China), followed by protein detection via a Western blot assay with His-tagged monoclonal antibodies [32, 33]. Further, pGEX-4t-1 (no gene fragment inserted) was incubated with the putative TBDRs, and this was used as the negative control to exclude nonspecific binding of prey proteins to the beads or to GST itself.

### Iron uptake test

To verify whether the four TBDRs are directly involved in iron uptake, we determined the Fe concentrations in the WT, mutant, and complemented strains of DE205B using an iron colorimetric assay kit (Elabscience Biotech, Wuhan, China, catalogue no. E-BC-K139-S). The WT, mutant (DE205BΔ*ireA*, DE205BΔ*tonB*, DE205BΔ*0007*, DE205BΔ*0008*, DE205BΔ*2235*), and complemented (DE205BΔ*ireA*/ireA*, DE205BΔ*tonB*/tonB*, DE205BΔ*0007*/0007*, DE205BΔ*0008*/0008*, DE205BΔ*2235*/2235*) strains were incubated overnight in 5 mL of LB medium supplemented with antibiotics, as appropriate. The cultured strains were then centrifuged (5000 rpm, 10 min), and the supernatant was discarded. The obtained cell pellets were washed twice with PBS containing 200 M 2,2′-dipyridyl, and the OD_600_ was adjusted to 1. Then, 1000 μL of the suspension was inoculated into 100 mL of LB media. Briefly, the strains were grown to the log phase and washed three times with 0.9% NaCl. Cell pellets were subsequently obtained and suspended in PBS, and the cells were lysed using an ultrasonic cell disrupter system (Thermo Fisher Scientific, USA) to release intracellular iron. Finally, impurities were removed by centrifugation (5000 rpm, 10 min); a sample of the supernatant was used for iron content determination [34]. Deionized water (0.5 mL), iron standard stock solution (0.5 mL), and sample (0.5 mL) were individually mixed with a chromogenic agent (1.5 mL), boiled for 5 min, and then centrifuged at 2300 rpm for 10 min; the supernatant was subsequently collected. According to the manufacturer’s instructions for the iron colorimetric assay kit, iron content was estimated by measuring the OD at 520 nm (OD_520_) of the supernatant. The following formula was used:

$$ \begin{aligned} {\text{Fe concentration }}\left( {\upmu{\text{g}}/{\text{L}}} \right)\, = \, & \left( {{\text{sample OD}}_{ 5 20} \, - \,{\text{deionized water OD}}_{ 5 20} /{\text{iron standard stock solution OD}}_{ 5 20} \,} \right)\, \\ & & {\kern 1pt} {\kern 1pt} {\kern 1pt} - \,{\text{deionized water OD}}_{ 5 20} \, \times \,{\text{standard iron concentration }}\left( { 2000 \upmu{\text{g}}/{\text{L}}} \right). \\ \end{aligned} $$

### Statistical analyses

Statistical analyses were performed in GraphPad Prism 7.0 using unpaired t-tests [35]. In the figures, the error bars indicate the standard deviations, “*” represents *P* < 0.05, “**” represents *P* < 0.01, and “***” represents *P* < 0.001. qRT-PCR data from three individual experiments were used to determine the differences (fold change) in gene transcription levels. Similarly, in the growth curve and iron uptake analyses, each reaction was performed three times to overcome any experimental errors.

## Results

### Iron depletion upregulated the expression levels of the genes encoding the putative TBDRs

**Figure 1:**
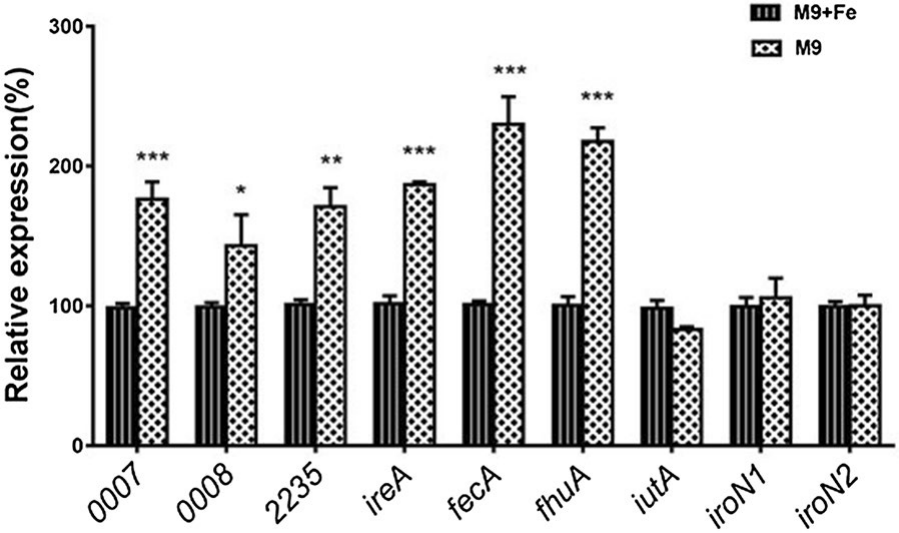
**Expression of the iron uptake-related genes in M9 media**. The gene expression levels of the iron uptake-related genes in iron-rich or iron-depleted M9 media were tested by qRT-PCR. The relative gene expression levels were calculated using the 2^−△△Ct^ method; the values are expressed as percentages.

The expression levels of all four genes were significantly upregulated under iron-depleted conditions compared with iron-rich conditions; the expression levels of *0007*, *0008* and *2235* were upregulated 1.76 times (*P* < 0.001), 1.43 times (*P* < 0.05), and 1.71 times (*P* < 0.01), respectively. The *ireA* gene was the most upregulated (1.87 times, *P* < 0.001) (Figure 1). In addition, the expression levels of the confirmed iron-uptake genes were upregulated under iron-depleted conditions compared to iron-rich conditions: the expression levels of *fecA* and *fhuA* were significantly upregulated (*P* < 0.01), but those of *iutA*, *iroN1*, and *iroN2* were not significantly changed (Figure 1).

### Iron depletion decreased the growth rate of the mutant strains

**Figure 2:**
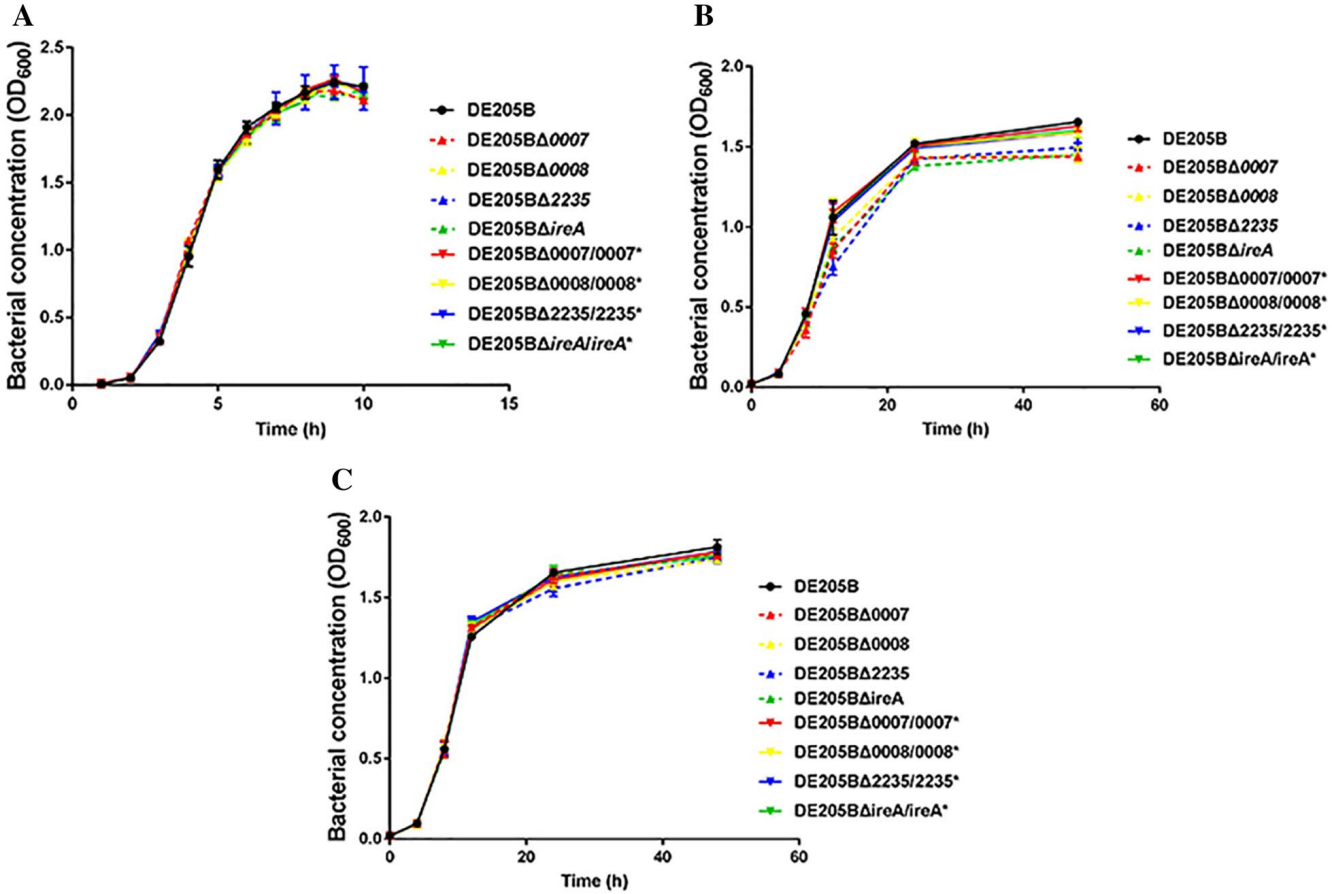
**The growth curves of the different strains cultured in LB and iron-depleted M9 media**. The growth curves of the WT, mutant (DE205BΔ*0007*, DE205BΔ*0008*, DE205BΔ*223*5, DE205BΔ*ireA*) and complemented (DE205BΔ*0007*/0007*, DE205BΔ*0008*/0008*, DE205BΔ*2235*/2235*, DE205BΔ*ireA*/ireA*) strains cultured in LB, iron-depleted and iron-rich M9 media. Bacterial growth was estimated by measuring the optical density at 600 nm (OD_600_). **A** LB medium; **B** iron-depleted M9 medium; **C** iron-rich M9 medium.

Growth curves of the WT and mutant strains cultured in LB, iron-depleted M9 and iron-rich M9 media were constructed. The obtained results indicated that the growth rates of the mutant strains (DE205BΔ*ireA*, DE205BΔ*0007*, DE205BΔ*0008* and DE205BΔ*2235*) were comparable to that of the WT strain in LB medium (Figure 2A). Under iron-depleted conditions, the growth rates of the mutant strains were slightly lower than that of the WT strain (Figure 2B). However, under iron-rich conditions, the growth rates of the mutant strains were comparable to that of the WT strain (Figure 2C).

### Compensatory expression of other iron uptake-related genes was significantly upregulated in the mutant strains

**Figure 3:**
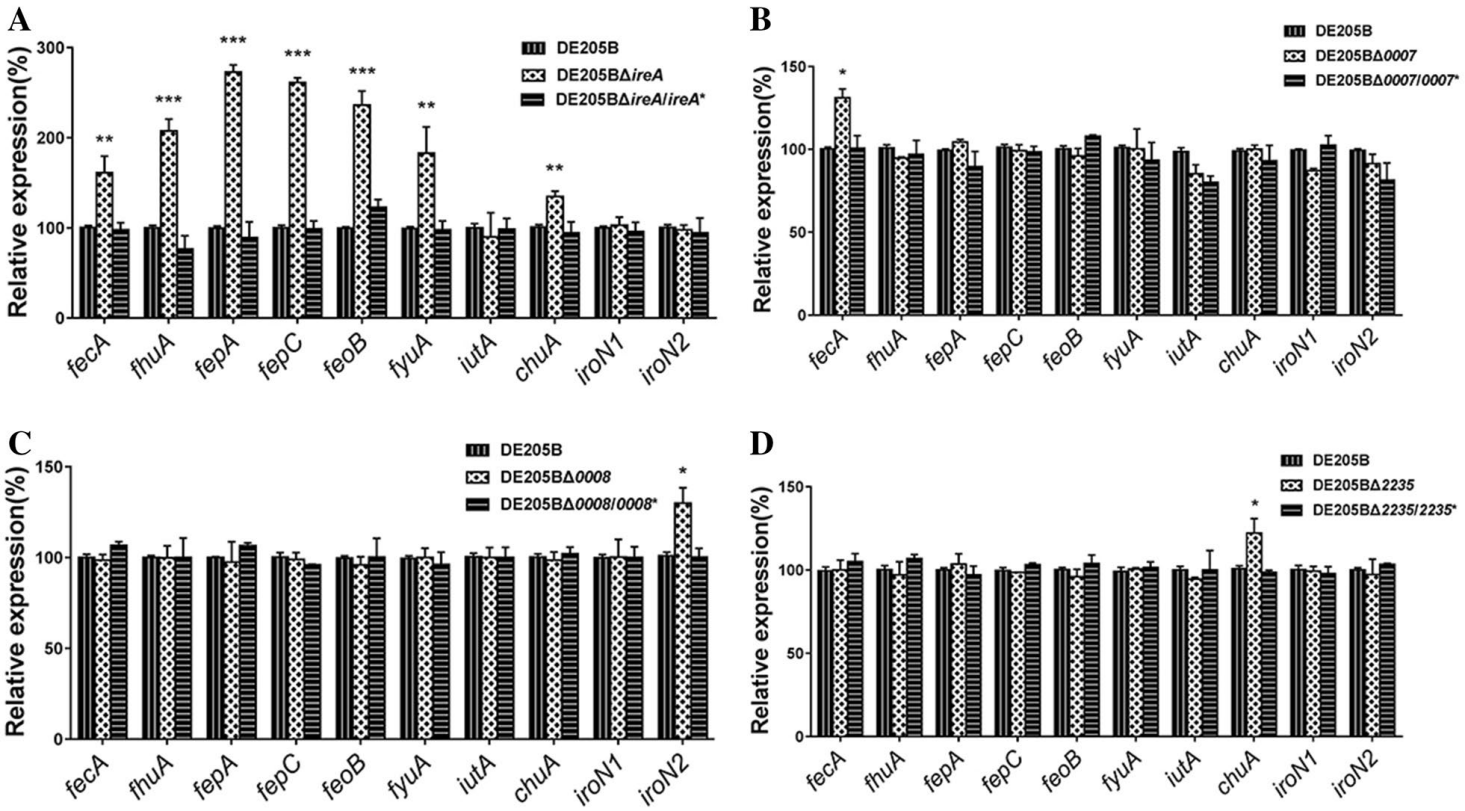
**The expression levels of the iron uptake-related genes**. The expression levels of the iron uptake-related genes (*fecA*, *fhuA*, *fepA*, *fepC*, *feoB*, *fyuA*, *iutA*, *chuA*, *iroN1*, *iroN2*) were tested by qPCR. **A** The expression levels of the iron uptake-related genes in the WT strain, DE205BΔ*ireA*, and DE205BΔ*ireA*/ireA*. **B** The expression levels of the iron uptake-related genes in the WT strain, DE205BΔ*0007*, and DE205BΔ*0007*/0007*. **C** The expression levels of the iron uptake-related genes in the WT strain, DE205BΔ*0008*, and DE205BΔ*0008*/0008*. **D** The expression levels of the iron uptake-related genes in the WT strain, DE205BΔ*2235*, and DE205BΔ*2235*/2235*. The relative gene expression levels were calculated using the 2^−△△Ct^ method; the values are expressed as percentages.

Most iron uptake-related genes in DE205BΔ*ireA*, including *fecA*, *fhuA*, *fepA*, *fepC*, *feoB*, and *fyuA*, were significantly upregulated (*P* < 0.05); the expression levels of all these genes were restored to WT levels in the complemented strain DE205BΔ*ireA*/ireA* (Figure 3A). In contrast, relative to those of the WT strain, the expression levels of *fecA*, *chuA*, and *iroN2* were higher only in DE205BΔ*0007*, DE205BΔ*0008*, and DE205BΔ*2235*, respectively (Figures 3B–D).

### IreA, 0007, 0008, and 2235 are TBDRs

**Figure 4:**
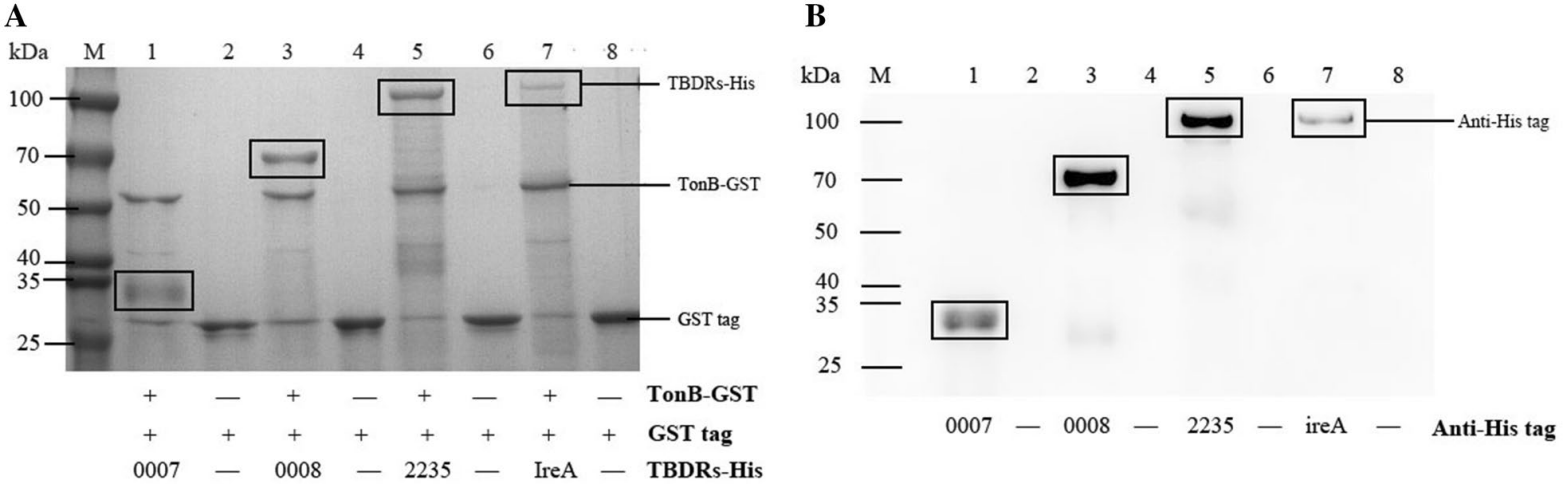
**GST pulldown assay**. In vitro GST pulldown assays indicating interactions between TonB and the putative TBDRs (IreA, 0007, 0008, 2235) were performed. Incubation of pGEX-4t-1 (no gene fragment inserted) with a putative protein was used as a negative control. **A** SDS-PAGE. TBDRs with the His tag (TBDRs-His) are highlighted using black boxes; **B** Western blot. All His tags are marked with black boxes. M: protein marker (kDa); 1: TonB + 0007; 2: pGEX-4t-1 + 0007; 3: TonB + 0008; 4: pGEX-4t-1 + 0008; 5: TonB + 2235; 6: pGEX-4t-1 + 2235; 7: TonB + IreA; and 8: pGEX-4t-1 + IreA.

Protein–protein interactions between the four putative TBDRs and TonB were analysed in vitro. The SDS-PAGE results showed that these proteins specifically bound to TonB; further, the Western blot assay results revealed the presence of a His tag on the TBDRs. These findings confirmed that TonB positively interacted with all four putative TBDRs (Figures 4A, B).

### Iron uptake by DE205BΔ*ireA* increased and that by DE205BΔ*tonB* decreased

**Figure 5:**
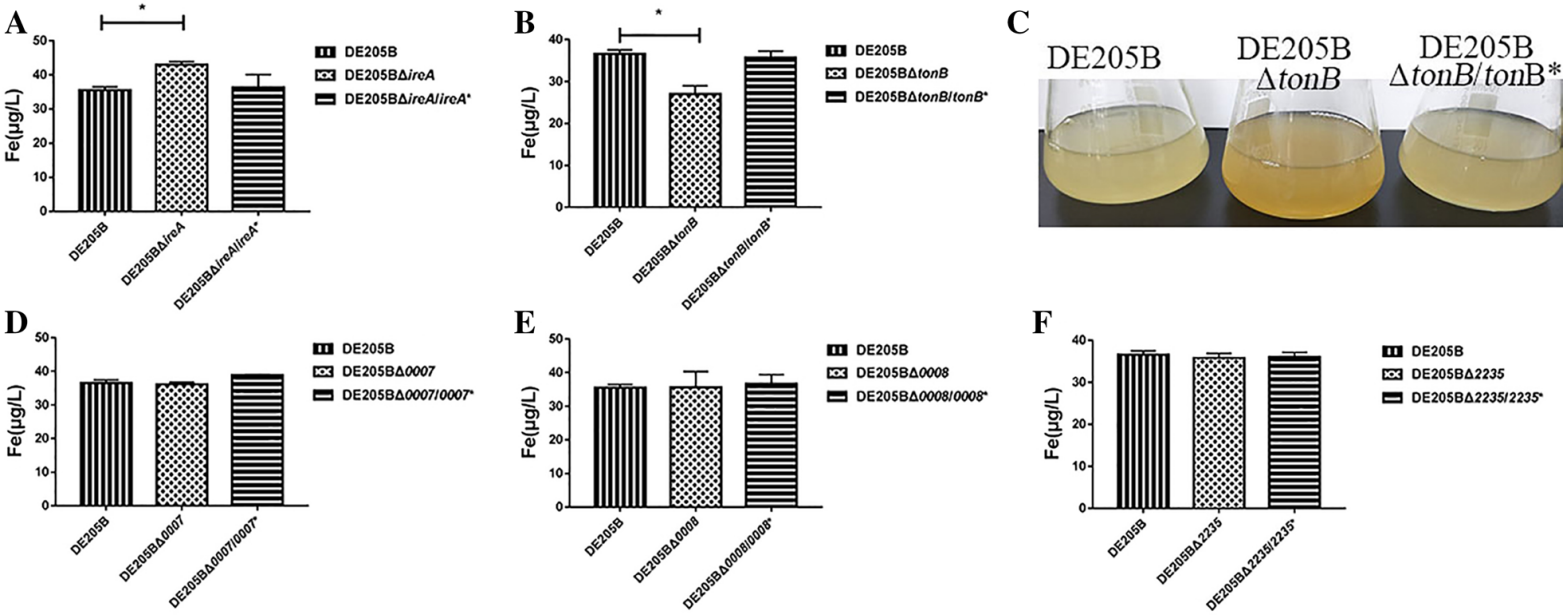
**The cellular Fe contents of the WT, mutant, and complemented strains**. The different strains were cultured in LB medium to the log phase. The cellular Fe contents of the WT, mutant, and complemented strains were determined using an iron colorimetric assay kit. **A** The cellular Fe contents of the WT strain, DE205BΔ*ireA*, and DE205BΔ*ireA*/ireA*; **B** The cellular Fe contents of the WT strain, DE205BΔ*tonB*, and DE205BΔ*tonB*/tonB*; **C** The culture medium used to grow DE205BΔ*tonB* turned red; **D** The cellular Fe contents of the WT strain, DE205BΔ*0007*, and DE205BΔ*0007*/0007*; **E** The cellular Fe contents of the WT strain, DE205BΔ*0008*, and DE205BΔ*0008*/0008*; **F** The cellular Fe contents of the WT strain, DE205BΔ*2235*, and DE205BΔ*2235*/2235*.

We found that at the mid-log phase of growth, Fe uptake by DE205BΔ*ireA* (Figure 5A) increased (*P* < 0.05) compared to that by the WT strain, whereas Fe uptake by DE205BΔ*tonB* (Figure 5B) decreased (*P* < 0.05). Fe uptake by the complemented strains was restored to normal. There were no significant differences in Fe uptake between the *0007*, *0008*, and *2235* mutant strains and the WT strain (Figures 5D–F). In addition, compared to the medium for the WT and complemented strains, the medium in which DE205BΔ*tonB* was cultured turned red (Figure 5C).

### The TBDRs are regulated by *fur*

**Figure 6:**
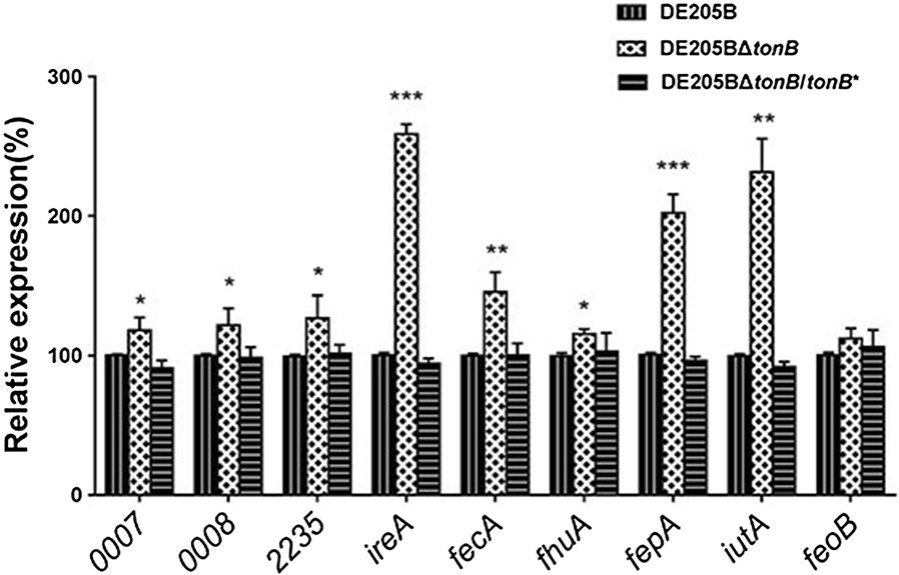
**The expression levels of the genes encoding the TBDRs under iron-depleted conditions**. The expression levels of the genes encoding the TBDRs under iron-depleted conditions, including *0007*, *0008*, *2235*, *ireA*, *fecA*, *fhuA*, *fepA*, *iutA*, and *feoB*, were tested by qRT-PCR using the WT strain, DE205BΔ*tonB*, and DE205BΔ*tonB*/tonB*. The relative gene expression levels were calculated using the 2^−△△Ct^ method; the values are expressed as percentages.

**Figure 7:**
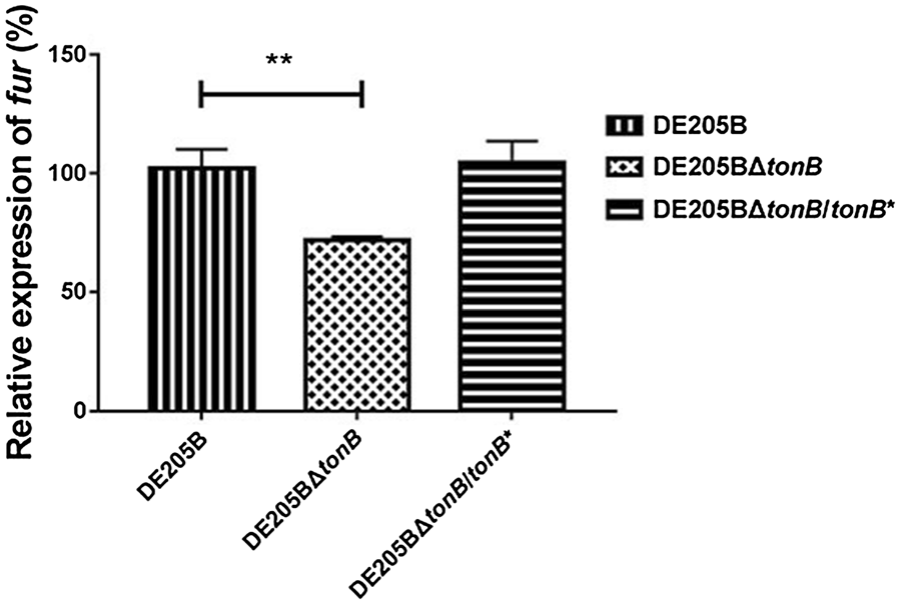
**The expression level of** ***fur*** **in iron-depleted M9 media**. The three columns represent the relative gene expression levels of *fur* in the WT strain, DE205BΔ*tonB*, and DE205BΔ*tonB*/tonB*. The expression level of *fur* in the mutant strain was significantly downregulated relative to that of the WT strain (*P* < 0.01).

The expression levels of the genes encoding the TBDRs were examined by qRT-PCR in the WT strain, DE205BΔ*tonB*, and DE205BΔ*tonB*/tonB* under iron-depleted conditions. The *ireA* expression level in DE205BΔ*tonB* was significantly upregulated (*P* < 0.01); furthermore, the expression levels of *0007*, *0008*, and *2235* were slightly upregulated (Figure 6). In addition, we observed that the expression level of the well-known TBDR *fepA* was significantly increased (*P* < 0.01), consistent with the results for *ireA.* Given that the TBDRs are under the control of the ferric uptake regulator *fur*, the expression levels of *fur* in the WT strain and DE205BΔ*tonB* under iron-depleted conditions were examined. qRT-PCR showed that relative to that of the WT strain, the expression level of *fur* in DE205BΔ*tonB* was downregulated (*P* < 0.01), indicating that the expression of the genes encoding the TBDRs was regulated by *fur* in the mutant strain DE205BΔ*tonB* (Figure 7).

**Figure 8:**
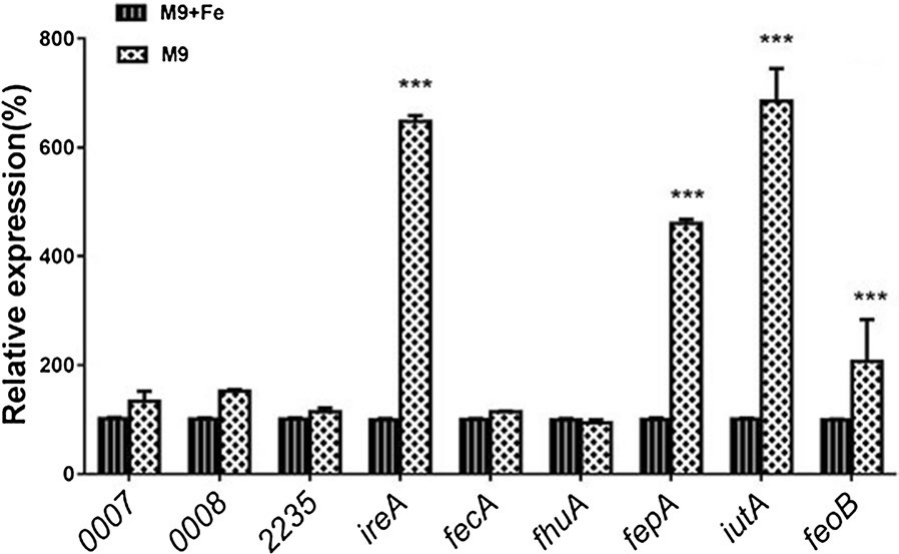
**The expression levels of the genes encoding the TBDRs in the mutant strain DE205BΔ*tonB*** **cultured in M9 media**. The expression levels of the genes encoding the TBDRs were tested by qRT-PCR under both iron-rich and iron-depleted conditions. The relative gene expression levels were calculated using the 2^−△△Ct^ method; the values are expressed as percentages.

The expression levels of the genes encoding the four TBDRs in DE205BΔ*tonB* were further tested under iron-rich or iron-depleted conditions using qRT-PCR. The obtained results indicated that the expression level of *ireA* was upregulated 6.5 times (*P* < 0.05) under iron-depleted conditions compared to iron-rich conditions and that the expression levels of *0007*, *0008*, and *2235* were also slightly upregulated (Figure 8). The expression levels of the genes encoding known TBDRs, including *fepA*, *iutA*, and *feoB*, were also significantly increased (*P* < 0.05).

## Discussion

Here, six putative TBDRs were predicted, but deletion or mutant strains could be obtained for only four genes encoding TBDRs (*ireA*, *0007*, *0008*, *2235*). Motif analyses of *ireA* sequences have helped reveal the C-terminal region of the TBDRs [12, 13], but experimental verification is lacking. Herein, IreA and TonB were expressed in vitro, and their interaction was determined via GST pulldown assays, which confirmed that *ireA* is a TBDR. In addition to *ireA*, three genes (*0007*, *0008*, and *2235*) were confirmed to encode TBDRs; however, compared to the expression levels of *0007*, *0008*, and *2235*, that of *ireA* was the most upregulated in response to iron depletion. Moreover, in the *ireA* mutant strain DE205BΔ*ireA*, iron uptake was increased, consistent with the excessive compensatory expression of other iron uptake-related genes. These findings are similar to those reported for the known TBDR *fepA*, indicating that *ireA* might function similarly to *fepA* [17].

TBDRs transport ferric siderophores on the OM with a lack of energy, relying on TonB to transduce energy from the proton motive force of the ExbB–ExbD complex in the inner membrane [36, 37]. In this study, a *tonB* mutant strain was constructed to analyse the effect of *tonB* deletion on TBDRs. The obtained results indicated that iron uptake by this mutant strain was significantly decreased; the medium used for culturing DE205BΔ*tonB* turned red as the free iron in the culture medium was oxidized. In addition, the expression level of *ireA* in DE205BΔ*tonB* was significantly upregulated and that of *fur* was downregulated. Due to severe iron uptake defects, an iron-depleted environment was created, causing *fur* to upregulate the expression of the TBDRs in DE205BΔ*tonB*, which implies that the TBDRs are regulated by *fur* and not directly affected by the presence or absence of *tonB*.

*ireA* was initially found in ExPEC, predicted to be a TBDR, and believed to play a role in the colonization of *E. coli* by functioning as a receptor on the OM [13]. In this study, *ireA* was indeed shown to be a TBDR. Our previous research has proven that *ireA* also plays a role in adhesion and stress resistance in APEC [13, 21]. Adhesion is the first step in APEC colonization, and the development of a receptor antagonist could protect against the establishment of a bacterial infection. As a multifunctional receptor, IreA may have potential for vaccine development. Each TBDR is composed of a 22-strand β-barrel and an N-terminal TonB box domain with which TonB interacts [38–40]. TonB interacts in vivo with the TonB box of TBDRs (FepA, FhuA, FecA) to form disulphide bonds and eventually transport iron into cells. TBDRs specifically recognize iron carriers. However, the TonB box domain of IreA has not yet been identified, and the iron siderophore structure to which it binds has not been studied either. Future research should focus on investigating these issues.

## Supplementary information

**Additional file 1:** Primers used in this study.

## Data Availability

The datasets analysed during the current study are available upon request from the corresponding author.

## Abbreviations

APEC: avian pathogenic *Escherichia coli*
OM: outer membrane
LB: Luria–Bertani medium
OD_600_: optical density at 600 nm
qRT-PCR: quantitative real-time polymerase chain reaction
GSH: glutathione, r-glutamyl cysteine + glycine
GSSH: oxidized glutathione
GST: glutathione-S-transferase
WT: wild-type
