# Supplementary material for: Identification of ireA, 0007, 0008, and 2235 as TonB-dependent receptors in the avian pathogenic Escherichia coli strain DE205B
Source: Vet Res. 2020 Jan 23;51:5. doi: 10.1186/s13567-020-0734-z (PMC6979363; doi:10.1186/s13567-020-0734-z)
Supplement: Supplementary file 1 — Additional file 1: Primers used in this study. [file 13567_2020_734_MOESM1_ESM.doc]

**Additional file 1 Primers used in this study**

| Primers | Sequence (5′–3′) | Gene | Reference | Usages |
| --- | --- | --- | --- | --- |
| Q*0007*-F | GCAGCGACCAGTCATTCTTC | *0007* | This study | a |
| Q*0007*-R | CATTCGTGACGACACTGACC |  |  |  |
| Q*0008*-F | GGGATGGGATATGCAGGGAA | *0008* | This study | a |
| Q*0008*-R | GCTGCTGGCATCCATACTTT |  |  |  |
| Q*2235*-F | CATTGTTCCAGACCGACACC | *2235* | This study | a |
| Q*2235*-R | CTGCGATAGGTCGCATCAAG |  |  |  |
| Q*ireA*-F | AAACATGGGATGGCGTACTT | *ireA* | This study | a |
| Q*ireA*-R | AATCAATGGGCCTGACAGATAG |  |  |  |
| Q*fecA*-F | AAATCGACATCGGCAACTGG | *fecA* | This study | a |
| Q*fecA*-R | CGAACGAGCCTTCAGTGTTT |  |  |  |
| Q*fhuA*-F | CCGTTGATACCCAGTTGCAG | *fhuA* | This study | a |
| Q*fhuA*-R | CGGGTCTTTCGCATTGAAGT |  |  |  |
| Q*iutA*-F | AGAAATTTGGCGGCTGGTTT | *iutA* | This study | a |
| QiutA-R | TTCAGCGTACCAGTTCCCAT |  |  |  |
| Q*iroN1*-F | GTTACCGAAAGCCTGGCAAA | *iroN1* | This study | a |
| Q*iroN1*-R | ACCGCCGGATAATCCTTCAT |  |  |  |
| Q*iroN2*-F | ACCGCCAGATCGATATTCGT | *iroN2* | This study | a |
| Q*iroN2*-R | CCGCGGATCACTTCAATACG |  |  |  |
| Q*fepA*-F | CAATGCGCCAGAACATAAAGAG | *fepA* | This study | a |
| Q*fepA*-R | TGTCGAGGTTGCCATACAAG |  |  |  |
| Q*fepC*-F | TCGTTACGCCAGCCATTT | *fepC* | This study | a |
| Q*fepC*-R | TGCAGCGCAGACCATAAA |  |  |  |
| Q*feoB*-F | GCACTCTTTGTGCATGGTATTC | *feoB* | This study | a |
| Q*feoB*-R | TGGCAGCACGGTGTTAAT |  |  |  |
| Q*fyuA*-F | ATGCCTATGTGGGATGGAATG | *fyuA* | This study | a |
| Q*fyuA*-R | CCAGTCATCGGTGGTGTATTT |  |  |  |
| Q*chuA*-F | TAGGCCACATCAAGGCTAAAC | *chuA* | This study | a |
| Q*chuA*-R | CGGCGACAACTATGTCGTATAA |  |  |  |
| Q*fur*-F | GTAACGCTTCCTCGCTTGAA | *fur* | This study | a |
| Q*fur*-R | AAATCTTCCGCACTGACGTG |  |  |  |
| Q*dnaE*-F | ATGTCGGAGGCGTAAGGCT | *dnaE* | [21] | a |
| Q*dnaE*-R | TCCAGGGCGTCAGTAAACAA |  |  |  |
| *0007*Mu-F | TCAGAGTTGCATAAATAAAATGTTATGTTATAACATTTTAATACCAACATATGTTTTCGAGTGTAGGCTGGAGCTGCTTC | Upstream region of *0007* | This study | b |
| *0007*Mu-R | CGAACCAGGTGAGCCCCGAGCCTGTGCTGTGGTCACCACTGGCAATATTTCCCCACAGACCATATGAATATCCTCCTTAG | Downstream region of *0007* | This study |  |
| *0008*Mu-F | AGGCTCGGGGCTCACCTGGTTCGGTAAAACCGGAAAAACAGATGCGCTCCTTTCTGTCATGTGTAGGCTGGAGCTGCTTC | Upstream region of *0008* | This study | b |
| *0008*Mu-R | TGTCAGACAAAGAGCTAAGATATAGCCTGTATTTTTCATCATGATTTATTTCCTGAGAGGCATATGAATATCCTCCTTAG | Downstream region of *0008* | This study |  |
| *2235*Mu-F | CATAAATTTACAATTTCATTACCATGACGGTGTTTTCCCATTCTTAACCTGGCTTTTTTCGTGTAGGCTGGAGCTGCTTC | Upstream region of *2235* | This study | b |
| *2235*Mu-R | TGTGCCAGCAATACAGCCGATAAATAAAATACGCCCGGCAAATACCGGGCTTAAAGATAACATATGAATATCCTCCTTAG | Downstream region of *2235* | This study |  |
| *tonB*Mu-F | CGCGCCTTACCCGTTGAGTAATAATCAAAAGCCTCCGGTCGGAGGCTTTTGACTTTATGCGTGTAGGCTGGAGCTGCTTC | Upstream region of*tonB* | This study | b |
| *tonB*Mu-R | GATTGCTATTTGCATTTAAAATCGGGACCTGGTTTTTCTACTGAAATGATTATGACTTCACATATGAATATCCTCCTTAG | Downstream region of *tonB* | This study |  |
| *0007*-F | ATCCAATACGGGAGGTTAC | *0007* | This study | c |
| *0007*-R | GCCATAACTTAACTGATGGG |  |  |  |
| *0008*-F | AGCGACCAGTCATTCTTCT | *0008* | This study | c |
| *0008*-R | TCTTCACCGAAACGACTG |  |  |  |
| *2235*-F | CCGCCTTTATCCAGTTTG | *2235* | This study | c |
| *2235*-R | CGTTTGGTCATTCCAGATAG |  |  |  |
| *tonB*-F | CGCTGTATCTCCTCTGTTCT | *tonB* | This study | c |
| *tonB*-R | TCTGCTTGTGGTGGTGAA |  |  |  |
| k1 | CAGTCATAGCCGAATAGCCT | *kanR* | [21] | c |
| k2 | CGGTGCCCTGAATGAACTGC |  |  | c |
| *0007*Co-F | CATGATTACGAATTCGAGCTCATGTATATGAATGTAATCAGAACTGTCATTT | *0007* | This study | d |
| *0007*Co-R | CAGGTCGACTCTAGAGGATCCTTAAACCGTTTGTCTCTCCGGG |  |  |  |
| *0008*Co-F | CATGATTACGAATTCGAGCTCATGCGTAAAAGAGGTAATATCTATCAAAG | *0008* | This study | d |
| *0008*Co-R | CAGGTCGACTCTAGAGGATCCTTACCACTGATAACGGGTATAAAGACTG |  |  |  |
| *2235*Co-F | CATGATTACGAATTCGAGCTCATGAAGATTTTTTCCGTCCGAC | *2235* | This study | d |
| *2235*Co-R | CAGGTCGACTCTAGAGGATCCTTACTCAAATCGCCACGCAA |  |  |  |
| *tonB*Co-F | CATGATTACGAATTCGAGCTCATGACCCTTGATTTACCTCGCC | *tonB* | This study | d |
| *tonB*Co-R | CAGGTCGACTCTAGAGGATCCTTACTGAATTTCGGTAGTGCCGT |  |  |  |
| *0007*Exp-F | GATCTGGTTCCGCGTGGATCCATGGCGACCAGTCATTCTTCTATG | *0007* | This study | e |
| *0007*Exp-R | TCAGTCAGTCACGATGCGGCCGCTTAAACCGTTTGTCTCTCCGGG |  |  |  |
| *0008*Exp-F | GCCATGGCTGATATCGGATCCATGAACAAGGAAAAACCTGCAGC | *0008* | This study | e |
| *0008*Exp-R | TGGTGGTGCTCGAGTGCGGCCGCTTACCACTGATAACGGGTATAAAGACTG |  |  |  |
| *2235*Exp-F | GCCATGGCTGATATCGGATCCATGGCTGATGAACAGACCATGA | *2235* | This study | e |
| *2235*Exp-R | TGGTGGTGCTCGAGTGCGGCCGCTTACTCAAATCGCCACGCAA |  |  |  |
| *ireA*Exp-F | GCTGATATCGGATCCGAATTCATGACCCTTGATTTACCTCGCC | *ireA* | This study | e |
| *ireA*Exp-R | GTGGTGGTGGTGGTGCTCGAGTTACTGAATTTCGGTGGTGCC |  |  |  |
| *tonB*Exp-F | GATCTGGTTCCGCGTGGATCCATGCAGGTTATTGAACTACCTGCG | *tonB* | This study | e |
| *tonB*Exp-R | TCAGTCAGTCACGATGCGGCCGCTTACTGAATTTCGGTAGTGCCGT |  |  |  |

Notes: a, used for RT-PCR; b, used for construction of mutants; c, used for identification of mutant strains; d, used for construction of complementary strains; e, used for GST pulldown assay
